# Nanotechnology for Nanophytopathogens: From Detection to the Management of Plant Viruses

**DOI:** 10.1155/2022/8688584

**Published:** 2022-10-03

**Authors:** Rachana Singh, Mohammad Kuddus, Pradhyumna Kumar Singh, Deki Choden

**Affiliations:** ^1^Amity Institute of Biotechnology, Amity University Uttar Pradesh, Lucknow Campus, Lucknow, Uttar Pradesh, India; ^2^Department of Biochemistry, College of Medicine, University of Hail, Hail, Saudi Arabia; ^3^Plant Molecular Biology and Biotechnology Division, Council of Scientific and Industrial Research-National Botanical Research Institute (CSIR-NBRI), Rana Pratap Marg, Lucknow, India; ^4^Sherubtse College, Royal University of Bhutan, Bhutan

## Abstract

Plant viruses are the most destructive pathogens which cause devastating losses to crops due to their diversity in the genome, rapid evolution, mutation or recombination in the genome, and lack of management options. It is important to develop a reliable remedy to improve the management of plant viral diseases in economically important crops. Some reports show the efficiency of metal nanoparticles and engineered nanomaterials and their wide range of applications in nanoagriculture. Currently, there are reports for the use of nanoparticles as an antibacterial and antifungal agent in plants and animals too, but few reports as plant antiviral. “Nanophytovirology” has been emerged as a new branch that covers nanobased management approaches to deal with devastating plant viruses. Varied nanoparticles have specific physicochemical properties that help them to interact in various unique and useful ways with viruses and their vectors along with the host plants. To explore the antiviral role of nanoparticles and for the effective management of plant viruses, it is imperative to understand all minute details such as the concentration/dosage of nanoparticles, time of application, application interval, and their mechanism of action. This review focused on different aspects of metal nanoparticles and metal oxides such as their interaction with plant viruses to explore the antiviral role and the multidimensional perspective of nanotechnology in plant viral disease detection, treatment, and management.

## 1. Introduction

Food security has always been the priority and important agenda around the globe to feed the large population [1]. Food sustainability is encountering a serious threat due to the manifestation of devastating infections followed by diseases in cultivated plants [2–4]. Majorly, crop infections are caused by plant pathogens such as bacteria [5], fungi [6, 7], and viruses [8–12]. Phytoviruses have been reported for several decades as the most contagious pathogens which cause drastic effects on plants. Various scientists working in plant virology have given critical reviews which have demonstrated that the heavy crop losses are due to virus diseases [13–24]. This loss can be measured in terms of both quantity and quality of produce [25]. The proper management of virus diseases of plants is always been a matter of great concern from farmers to horticulturists, manufacturers to consumers, and foresters.

For decades, nanotechnology has proved its potential for the development of effective formulations [26–28], but due to the paucity of commercial applications and its role in agriculture has not gained popularity, various studies showed the use of nanoparticles as insecticides, fungicides, or herbicides and discussed the nanoparticle formulations against a target pest. There are two mechanisms for the application of nanoparticles to safeguard plants: (i) nanoparticles themselves provide crop protection and (ii) nanoparticles used as carriers for existing pesticides, for example, the application of double-stranded RNA, can be done by spray application on foliar tissue or on roots or soaking of seeds [29, 30]. In this review, we present a focused discussion on different aspects of nanoparticles in plant viral disease detection, treatment, management, and their interaction with plant viruses. The new term is also given to this study called “nanophytovirology.”

## 2. Nanoparticles and Their Application against Plant Pathogens

**Figure 1:**
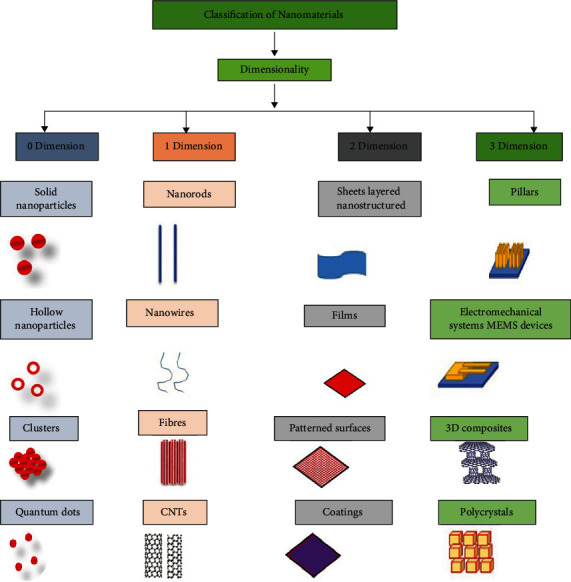
Diagrammatic representation of the classification of nanomaterials.

Nanoparticles (NPs) are small materials with nanosize ranging from 1 nm to 100 nm [31, 32] and are classified based on their shape or size and also (and most importantly) on their composition (Figure 1). The different class comprises metal NPs, ceramic NPs, polymeric NPs, and fullerenes. They show unique physiochemical properties due to their large surface-to-mass ratio, high reactivity, and unique interactions with biological systems [33]. Due to these unique properties and characteristics, they have gained attention in all fields from commercial to domestic, medical [34, 35] to agriculture [36], and environment [37, 38] to energy-based research [39–41]. The use of nanoparticles for sustainable agriculture was discussed in [31, 42, 43]. Different nanoparticles are used to design biosensors for the detection of plant disease, as the delivery vehicle for genetic materials [44], such as nanofertilizers and nanopesticides [28, 45].

The nanoparticles could be synthesized by three different methods: biological, physical, and chemical methods. Out of these, biological approaches are considered the best, due to their nontoxic effect, cost-effective, and environmentally friendly nature [46]. The method of synthesizing nanoparticles greatly influences their geometry and further affects the physiochemical properties like morphology, size, crystal structure, and dispersity. The biosynthetic method to synthesize nanoparticles by different methods and utilizing plants and microorganisms is very diverse. Preliminary microorganisms or plant extracts are exposed to metallic salts that in turn reduce the metal to its nanosize. The nanoparticles were further characterized and made available for further applications [47–49].

Numerous evaluations have been carried out that show the applications of nanoparticles related to plant diseases are either metalloids, metallic oxides, or nonmetals, involved in disease resistance as bactericide/fungicides or nanofertilizers (Table 1) [44, 50]. The metallic nanoparticles include pure metal and metal oxides [51]. The most popular metal nanoparticles comprise silver (Ag), gold (Au), platinum (Pt), nickel (Ni), and iron (Fe), and the metal oxide nanoparticle includes compounds such as TiO_2_, ZnO, MgO, CuO, Cu_2_O, Al_2_O_3_, NiO, and SnO_2_ [52].

## 3. Systematic Facets of Nanomaterials as Antiviral Agents

Phytoviruses are always being a challenge for farmers in terms of the production of crops and vegetables. There is a list of experiments that shows the application of different nanoparticles in bacterial and fungal diseases of plants; however, the focused study of nanoparticles on plant virus management is still in its preliminary stages, and the antiviral mechanisms of action of metal nanoparticles are not completely understood. The summary of published work and the available information concerning nanoparticles and plant viruses are gathered in Table 2.

**Figure 2:**
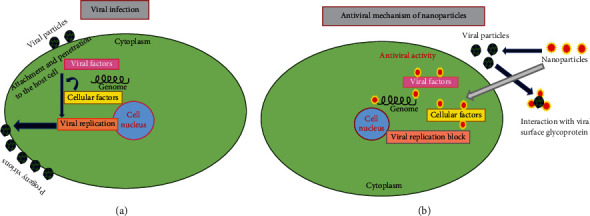
Diagrammatic representation of (a) virus particles infesting eukaryotic cell and (b) antiviral mechanics of metallic nanoparticle.

**Figure 3:**
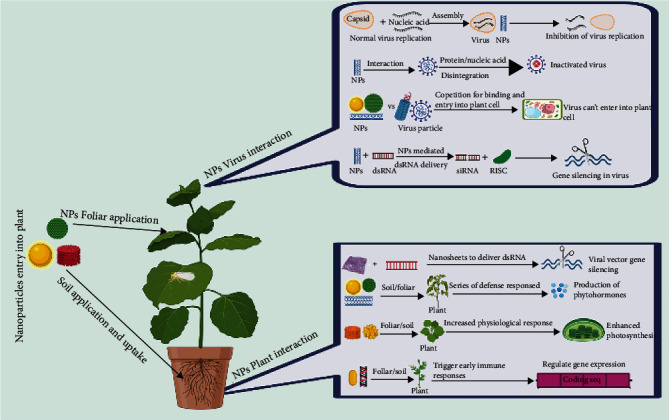
Mechanism of nanoparticle interaction with plant-virus system (created in http://BioRender.com).

**Figure 4:**
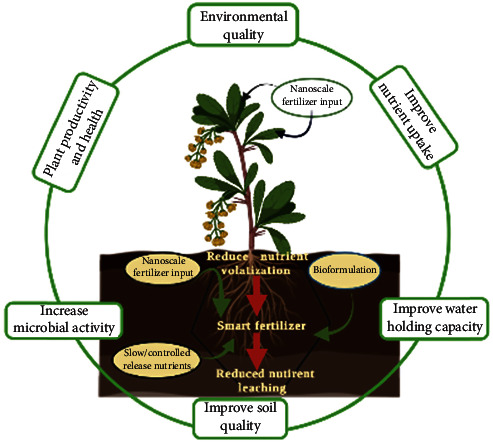
Diagrammatic representation of effects of potential smart (nano) fertilizers in soil-plant system (adapted from Calabi-Floody et al.) (created in http://Biorender.com).

**Figure 5:**
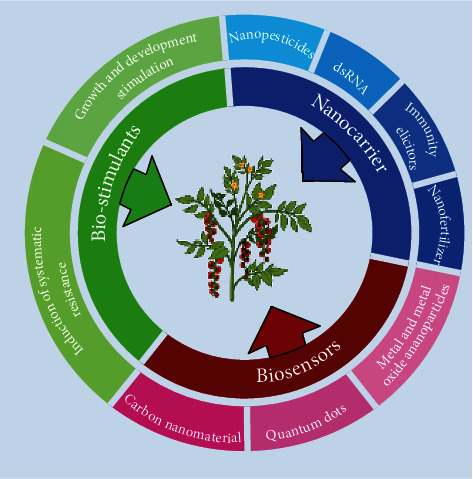
Various approaches of nanotechnology to attain antiviral protection in plants (created with http://BioRender.com).

The antiviral mechanism of NPs discussed in different studies and other different possible mechanisms (Figure 2) and the specific interactions between host (plant), vector (s), and pathogen (viruses) is summarized in (Figure 3, 4,and 5).

## 4. Antiviral Activity of Metallic Nanoparticles for Plants

To protect the plants from pathogen invasion, the nanomaterials can be applied directly either into the soil or to seeds or foliage. This direct application is similar to the use of chemical pesticides. However, direct application of nanoparticles to the soil directly affects microorganisms, especially nitrogen-fixing and mineral solubilizing which play a significant role in plant health and nutrition. Silver nanoparticles were the first to be used in plant disease management and showed their antimicrobial activity [53]. The nanoparticle's interface with bacterial and fungal pathogens is studied very well but with viral particles is still not explored well, although some researchers studied the antiviral and virucidal mode of action of silver nanoparticles (AgNPs) against plant viruses [54–56].

The antiviral mechanisms of metal nanoparticles are not very well understood, but the available studies could provide evidence of the mechanisms involved. The antiviral activity of MeNPs has been observed both in vitro and in vivo on different plants, and it is found to be effective against most of the RNA viruses. Various studies revealed that physical properties like size, shape, and surface area are the key factors to control the biological activity of any nanoparticle [57, 58]. Reports revealed that the antibacterial activity of AgNPs is size-dependent. The small size (10 nm) of AgNPs has shown more antibacterial affinity in comparison to larger ones [59]. Furthermore, the variable antimicrobial activity of nanoparticles is influenced by the shape of nanoparticles (spherical, rod-shaped, nanoshells, nanocages, nanowires, triangular, and dimensional).

The impact of AgNPs on the *Bean yellow mosaic virus* (BYMV) was studied and reported that the antiviral property of NPs is due to their ability to attach to the envelope glycoprotein of the virus. It binds the disulfide bond regions of the CD4-binding domain present in the envelope glycoprotein gp120 of yellow mosaic virus and prevents entry [54]. Apart from their interaction with the surface glycoprotein of the virus, AgNPs also interact with the nucleic acid of the virus to enter into the cell and complete their antiviral activity. This experiment was intended to compare the impact of the spray of AgNPs before infection, 24 h after infection, and at the time of inoculation. Another work was also evidenced the high attachment capacity of nanoparticles of different sizes (10 and 50 nm), to virus DNA and extracellular virions. It was also observed that the AgNPs inhibited the production of viral RNA and extracellular virions in in vitro conditions, verified by UV-Vis absorption assay [60] and also found to restrict the fusion of the viral membrane by hindering viral permeation into the host cell [61].

Sun and his coworkers compared the AgNPs and gold nanoparticles and found AgNPs superior when used for cytoprotective activity towards the virus. It was a general observation that various forms of silver nanoparticles can inactivate viruses by denaturing enzymes through different reactions with self-hydra, amino, carboxyl, phosphate, and imidazole groups [62–66]. Dougdoug et al. [67] experimented with the effectiveness of AgNPs as an antiviral agent against two plant viruses, *Potato virus Y* (PVY) and *Tomato mosaic virus* (ToMV) and observed the effect. Different concentrations (50, 60, and 70 ppm) of AgNPs was sprayed on the plants carrying both diseases and at 50 ppm a concentration of AgNP the striking decrease in disease severity and concentration of both viruses was observed. Furthermore, the transmission electron microscopy (TEM) analysis of the viral sap substantiated the binding of coated protein particles of the virus to AgNPs [67]. Furthermore, a study on *Sun-hemp rosette virus* (SHRV) indicated complete suppression of the viral disease when spraying with AgNPs at the concentration of 50 mg/L. The detailed result showed the binding of these NPs with virus coat protein and virus inactivation is due to inhibition of virus replication [68].

The antiviral effect of AgNPs was observed against *Tomato spot wilt virus* (TSWV) on *Chenopodium amaranticolor.* Plants sprayed 24 h after inoculation showed weak infection in comparison to plants sprayed before inoculation [69]. Similar result, reduction in virus concentration and disease percentage, was reported by El-shazly et al. on potato plants against *Tomato bushy stunt virus* (TBSV) [70], while *Cyamopsis tetragonoloba*, infected with *Sun-hemp rosette virus* (SHRV), displayed complete suppression of the disease and inactivation of virus replication [68]. The antiviral effect of ZnO and SiO_2_ NPs was studied on tobacco plants against TMV by Cai et al. Both NPs were applied on 3, 7, and 12 days before inoculation of virus. The plant treated 12-days before displayed an extreme antiviral effect by preventing TMV infection and spreading in new leaves [71]. Findings of his work suggest that the inhibition of TMV is due to interaction of metal NPs with envelope glycoproteins, resulting injury of TMV coat protein, and its aggregation. Hao et al. used Fe_2_O_3_ or TiO_2_NPs for pretreatment of tobacco plants for 21 days to check the antiviral properties against *Turnip mosaic virus* (TuMV). The results of the study showed a high decrease in viral proteins, in which the authors suggest could be related to the fact that the NPs interfered with either protein biosynthesis or posttranslational modification processes in the virus, and activated defense mechanisms [72]. Various reports confirmed its action against plant viruses as it successfully induced resistance to mosaic disease impeded by the virus in potato, alfalfa, cucumber, peanut, and snuff [72–74]. Malerba and Cerana reported various conceivable mechanisms of chitosan that precede the antimicrobial effects that includes disruption of the cell membrane, inhibition of toxin production and microbial growth, inhibition of H+ -ATPase activity, and preventing the synthesis of mRNA and proteins. Furthermore, their studies revealed the antiviral action of chitosan nanoparticles in bean plants infected with *bean mild mosaic virus*, tobacco plants infected with *tobacco necrosis virus* and *tobacco mosaic virus* [75].

Adeel et al. worked on *Nicotiana benthamiana* plants and given the treatment at different concentrations of titanium dioxide (TiO2) and silver (Ag) nanoparticles, C60 fullerenes, and carbon nanotubes (CNTs) at 100, 200, and 500 mg/L and observed for a 21-day foliar exposure before inoculation of *Tobacco mosaic virus* (TMV). Plants treated with CNTs and C60 (200 mg/L) exhibited normal phenotype, and viral symptomology was not evident at 5 days postinfection, whereas TiO2 and Ag NP-treated plants show no sign of virus infection suppression [76].

## 5. Nanotechnology in Diagnostics of Plant Viruses

Many molecular and serological techniques, viz., polymerase chain reaction (PCR), real time PCR, immunological assays such as Enzyme-linked immunosorbent assay (ELISA), and electrochemical immunoassay (ECIA), are being used for diagnostics and identification of plant viral pathogens [32, 77–80]. Although these techniques are efficiently and effectively detecting plant pathogens, it requires well-established laboratory settings with high-end equipment and chemical, well-trained/experienced individuals. With fast-developing technology, the hour demands to develop rapid, accurate, reliable, and miniaturized field-deployable devices which do not demand a very trained personnel [81]. The success of any management practice depends on the quick, early, and sensitive diagnostic of the infected material. Nanotechnology recommends major progress through quick and very sensitive pathogen probes in this area. Nanotechnology has gained a pace in the diagnostics of plant pathogens. Nanoparticles are being used as rapid diagnostic tools for the detection of bacterial, fungal, and nematodes, and very few reports [82, 83] are there in the diagnostics of plant virus disease. The use of superparamagnetic iron oxide nanoparticles has been used in medicine and water purification for decades [84, 85], but now, it has taken advancement, and its potential is being recently been explored in plant pathology. These magnetic nanoparticles adhere to the biological tissue and DNA, eventually facilitating the extraction and detection of the pathogen [86].

### 5.1. Biosensor-Based Detection

The device designed to detect the occurrence of any biological analyte, such as a biomolecule, a biological structure, or a microorganism, is known as biosensors. It consists of three parts: (i) a section that identifies the analyte and produces a signal, (ii) a signal transducer, and (iii) a reader device [87]. Various nanomaterials, basic metallic nanoparticles (carbon and gold nanoparticles), and nanospheres enhance the sensitivity of the assay when used in combination with aptamer-based detection systems.

### 5.2. Antibody-Based Detection

In recent years, various reports have manifested the antibody-based detection of plant viruses [88–90]. James and Lin et al. developed nanobased biosensors for the detection of the *Lettuce mosaic virus*, *Cowpea mosaic virus*, and *tobacco mosaic virus* with twofold increase of the sensitivity of detection in comparison to traditional methods of ELISA [89, 90]. Indirect ELISA was applied for the detection of *Cucumber mosaic virus* (CMV) by Jiao et al. This method of ELISA consists of three steps: (i) fixation of virus antigen on the surface, (ii) treatment with specific antibodies for the detection of the virus, and (iii) incubation with an enzyme and horse shoe peroxidase- (HRP-) labeled secondary antibody. The reaction was monitored by the mercury electrode. This electrochemical enzyme-linked immunoassay (ECEIA) sensor-based method showed four times higher sensitivity in the detection of CMV in comparison to the standard spectrophotometric ELISA. This was also observed with other plant viruses such as *Turnip mosaic virus* (TuMV), *Tobacco mosaic virus* (TMV), *Potato virus Y* (PVY), *Southern bean mosaic virus* (SBMV), and *Tomato aspermy virus* (ToAV).

In the case of immunosensors, self-assembled monolayers (SAM) were used for diagnostics of plant pathogens. In this method, gold electrodes are the most commonly used substrate for the detection of *Plum pox virus* (PPV) [91]. Later on, Jarocka et al. in 2013 applied the same method for the diagnostic of *Prunus necrotic ringspot virus* (PNRSV) and concluded that the biosensor has alike similarity as ELISA [92]. Another biosensor-based plant virus detection was discussed by Huang et al. [93]. He used the quartz crystal microbalance immune sensor that was based on SAMs for identification of *Maize chlorotic mottle virus* (MCMV). The sensitivity of the biosensor was found to be similar to ELISA with a detection limit of 250 ng/mL and showed high sensitivity with similar viruses such as *Wheat streak mosaic virus* (WSMV) [93]. Lateral flow immunoassay (LFIA), a type of optical immunosensor, was initially used by Tsuda et al. [94] for the detection of the *Tobacco mosaic virus* (TMV). Later on, this method was employed for the diagnostic of several other viruses, *Citrus tristeza virus* (CTV) [95], *Potato virus X* (PVX) [96], *Potato virus x* [97], *Potato virus Y* (PVY), *Potato virus M* (PVM), and *Potato virus A* (PVA) with a reported sensitivity of 2 ng/mL.

An immunoassay is reported to be developed for the detection of multiple substances such as biomarkers and plant pathogens that function based on fluorescence-loaded magnetic microspheres and fluorophore antibodies [98, 99]. A study has been conducted using specific antibodies for plant viruses, *Chilli vein-banding mottle virus* (CVbMV), *Watermelon silver mottle virus* (WSMoV), and *Melon yellow spot virus* (MYSV) [100]. Although the techniques have shown high sensitivity for detection along with the capacity of multiple detections in a single assay, they did not become very popular due to the complexity of assays and fluorescent readers. Various reports mentioned the use of label-free biosensors, based on SPR, developed for the detection of CMV, TMV, and *Lettuce mosaic virus* [101–104] and for orchid viruses, *Cymbidium mosaic virus* (CymMV) or *Odontoglossum ringspot virus* (ORSV) [90].  Table 3 summarizes the application of different biosensors for the detection of various plant viruses.

### 5.3. Plant Virus Detection Based on Quantum Dots (QD)

Quantum dots (QD) are small semiconductor nanocrystals that have been used for the construction of biosensors [105]. It has been used for disease detection as it consists of a unique optical property that is used in fluorescence resonance energy transfer (FRET) [106]. Rad et al. used this approach for the detection of phytoplasma disease known as *Witches' broom disease of lime* (WBDL) caused by *Candidatus Phytoplasma aurantifolia* [107]. The consistent result with 100% specificity and sensitivity was achieved by this approach for approximately 5 *Candidatus Phytoplasma aurantifolia* per *μ*L. This technique was applied to detect *Rhizoctonia*, the disease vector of the *Beet necrotic yellow vein virus* (BNYVV) [108].

## 6. Metal Nanoparticles as Biostimulants in Virus-Infected Plants

Biostimulants are substances that enhance the physiological process of plants and promote growth, development, and defense responses. When applied directly to plants or seeds, they cannot be considered pesticides or nutrients [109]. The positive or negative effect of nanoparticles on the plant is based on the type of nanoparticles and the condition of the plant [110, 111]. Healthy tobacco plants were studied for the effect of SiO_2_, Fe_2_O_3_, and ZnO nanoparticles and observed to have increased growth [112, 113]. When the effect of NiONPs was observed on the virus-infected cucumber plants by foliar spray and soil drench, it showed an increased number of leaves along with higher fresh and dry weight [114]. The tobacco plant infected with *Turnip mosaic virus* was being treated with foliar spray of TiO2 and FeO3 with the concentration of 50 mg/L and observed with enhanced fresh and dry weight, whereas no effect was observed with the treatment of 200 mg/L in comparison to nontreated plants [115]. When the *Potato virus Y*-infested tubers were treated with AgNPs, they have shown improved quality parameters in comparison to infected but not treated plants. The reason may be the provocation of resistance or the effect of nanoparticles on virus entry [70, 113].

## 7. NPs as an Option to Control of Plant Viral Pathogens

### 7.1. Application of NPs in Plant Defense Induction and Viral Repression

#### 7.1.1. Antioxidant System

Under stress conditions (biotic or abiotic), the plant response is observed by increased reactive oxygen species (ROS) that limits the entry of the pathogen and its dissemination and stimulates local and systemic defense responses [71]. When the ROS level increases than the threshold, oxidative stress is being produced and this interrupts the steadiness between ROS and antioxidants. The role of antioxidants in plants is to counterpoise the antioxidants effect. Superoxide dismutase (SOD) acts as the initial boundary of defense and coverts the O_2_ into water and H_2_O_2_ [113, 114]. The enzymes like catalase, ascorbate peroxidase, and guaiacol peroxidase make antioxidant systems [113]. The type of metal nanoparticles, their concentration, and the culture type define the interaction of metal nanoparticles with cellular redox homeostasis and alter the incident of oxidative stress inducing or reducing it [114]. The foliar application of Fe_3_O_4_ NPs to tobacco leaves resulted in enhanced production of ROS, which indicates the stimulation of resistance against the virus in tobacco [71]. When cucumber plants were treated with SiO_2_ NP, they displayed the expression of pox and pal genes a day after inoculation of PRSV [116]. A similar observation was reported, with increased pod gene expression, when cucumber plants were treated with NiO NPs, after four days of CMV inoculation [112]. The AgNP-treated tomato plants when inoculated with TMV and PVY revealed a major increase in the activity of enzymes such as polyphenol oxidase and antioxidant enzyme POD [67, 117].

#### 7.1.2. Plant Hormones and Pathogenesis-Related Proteins

Plant hormones play important roles in the defense mechanism of the plant. The phytohormones like salicylic acid (SA), jasmonic acid (JA), and ethylene are the key factors to regulate pathways involved in the defense mechanism and induce appropriate responses. The other phytohormones which can modulate plant defense and responses are auxins, cytokinins, gibberellin, abscisic acid, brassinosteroids, and strigolactone. Different hormonal pathways are up- or downregulated in different types of stress. Nanoparticles have been shown to stimulate hormonal balance in plants [110]. Various studies and discussions concluded that the expression of any particular plant hormone is completely dependent on the particular interaction of plant and metal nanoparticles together with the dose and time of application. Vincovi'c et al. reported that treatment of *Capsicum annum* L plants with AgNPs increases cytokinin [117]. Tobacco plants infested with TMV, when given the treatment of Fe_2_O_3_ and TiO_2_ NPs, influence the levels of zeatin, ribose (ZR), abscisic acid, and brassinosteroid (BR) phytohormones [115]. When the treatment of similar nanoparticles was given to tobacco, plants infected with TuMV showed an enhanced level of BR and ZR, but the decrease in ABA concentration was observed. Various other reports suggest that treatment of ZnO and SiO_2_ [111] to uninfected tobacco plants upregulated salicylic acid- (SA-) induced pathogenesis and a similar effect was reported for Fe_3_O_4_ NPs [111].

## 8. Conclusion

Nanophytovirology is a very promising field towards sustainable crop protection against viruses. The different nanoparticles and their applications have tremendous potential to deal with plant virus disease-related problems. Among plant viruses, DNA plant viruses specially geminiviruses [118] are a continuous threat to farmers and cause a serious threat to the crops [12, 119]. It consists of a very wide host range, with varied symptoms. Geminivirus constitutes a major and rapidly emerging group [120, 121] of circular, single-stranded plant viruses. Various countries like the United States, Africa, India, and Pakistan have reported large crop losses due to geminivirus infection, worth several million dollars [10, 122, 123]. Moreover, the effect of nanomaterials in the tripartite interaction of plant-viruses-vector is still not known. Although various roles and uses have already been studied, precise complementary methodologies are needed to establish so that a ready-to-use technology could be given to farmers without posing any risk to the environment or consumers. This additional information and knowledge are required to particularize the doses, the stage of the plant for application, and the particular type of NPs that can produce the greatest advantages. In addition, the effect of nanoparticles on the virus-vector relationship also needs to be explored, whether it is dose-dependent or stage-dependent. It is important to say that for sustainable management of phytoviruses, the multidisciplinary research is required with proper planning, development, and implementation of nanobased antiviral strategies.

## Figures and Tables

**Table 1:** Types of nanoparticles and their use against plant pathogens.

Type of nanoparticles	Application in plant pathology
Metalloids, metallic oxides, nonmetals, and their composites	Bactericide/fungicide nanofertilizers carrier for antimicrobials and genetic material
Carbon nanomaterials	Multiple uses
Single-walled or multiwalled nanotubes	Antimicrobial agents, carrier for antimicrobials, and genetic material
Graphene oxide sheet (reduced or oxide forms)	Antimicrobial agents, carrier for antimicrobials, and genetic material
Liposomes	The delivery vehicle for genetic or antimicrobial formulations
Nanobiosensor	Diagnostics, research tool
Quantum dots	Diagnostics, research tool

**Table 2:** Types of nanoparticles and their effect on different plant viruses.

Type of nanoparticle	Plant pathogen	Effect	References
AgNPs	*Sun-hemp rosette virus*	Complete suppression of disease	[68]
AgNPs	*Tobacco mosaic virus* (TMV)	Decreased infection	[41]
AgNPs	*Potato virus Y*	Resistance against virus infection	[56, 67]
AgNPs	*Tomato spotted wilt virus*	Decrease in infectivity and reduced local lesions	[69]
AgNPs	*Tomato mosaic virus*	Reduced disease severity and virus infection	[67]
Gold nanoparticles (AuNPs)	*Barley yellow dwarf virus*	Destroyed coat protein and eliminated virus infection	[124]
AuNPs	*Barley yellow mosaic virus*	Virus particles dissociated	[125]
Zinc oxide nanoparticles (ZnONPs)	TMV	Reduction in the TMV invasion speed	[71]
ZnONPs	*Cucumber mosaic virus (*CMV)	Significant reduction in severity and incidence of disease	[56]
Titanium dioxide NPs (TiO_2_NPs)	*Turnip mosaic virus*	Decreased viral replication and infection	[115]
Iron dioxide NPs (Fe_2_O_3_ NPs)	*Turnip mosaic virus*	Effectively limits viral replication and infection	[72]
Fe_2_O_3_ NPs	TMV		[71]
Silicon dioxide NPs (SiO_2_NPs)	TMV	Reduction in the speed of virus invasion	[71]
SiO_2_NPs	*Papaya ringspot virus*	Suppression of disease severity and virus accumulation	[116]
SiO_2_NPs	*Tomato yellow leaf curl virus*	Reduced disease severity and virus concentration	[126]
SiO_2_NPs	TMV	Suppression in the speed of virus invasion	[71]

**Table 3:** Different biosensors used for the detection of plant viruses.

Biosensors	Plant viruses	Detection limit
Antibody-based	*Cucumber mosaic virus*	0.5 ng/mL
Antibody-based	*Plum pox virus*	10 pg/mL
Antibody-based	*Prunus necrotic ringspot virus*	—
Antibody-based	*Maize chlorotic mottle virus*	250 ng/mL
Antibody-based	*Potato virus x*	2 ng/mL
Antibody-based	*Chilli vein bending mottle virus*	35.3 ng mL
Antibody-based	*Watermelon silver mottle virus*	—
Antibody-based	*Melon yellow spot virus*	—
Antibody-based	*Cymbidium mosaic virus*	48 pg/mL
Antibody-based	*Odontoglossum ringspot virus*	42 pg/mL
DNA-based	*Plum pox virus*	12.8 pg/mL
DNA-based	*Banana bunchy top virus*	15 ng/mL
DNA-based	*Banana streak virus*	50 fm

## References

[1] Kleve S., Booth S., Davidson Z. E., Palermo C. (2018). Walking the food security tightrope-exploring the experiences of low-to-middle income Melbourne households. *International journal of environmental research and public health*.

[2] Woolhouse M. E., Haydon D. T., Antia R. (2005). Emerging pathogens: the epidemiology and evolution of species jumps. *Trends in ecology & evolution*.

[3] Cleaveland S., Haydon D. T., Taylor L. (2007). Overviews of pathogen emergence: which pathogens emerge, when and why?. *Current Topics in Microbiology and Immunology*.

[4] Jones R. A. C. (2009). Plant virus emergence and evolution: origins, new encounter scenarios, factors driving emergence, effects of changing world conditions, and prospects for control. *Virus Research*.

[5] Sudheep N. M., Marwal A., Lakra N., Anwar K., Mahmood S., Singh D. P. (2017). Fascinating fungal endophytes role and possible applications. *Plant-Microbe Interactions in Agro-Ecological Perspectives*.

[6] Prajapat R., Marwal A., Jha P. N. (2013). Erwinia carotovora associated with potato: a critical appraisal with respect to Indian perspective. *International Journal of Current Microbiology and Applied Sciences*.

[7] Mahmood S., Lakra N., Marwal A., Sudheep N. M., Anwar K., Singh (2017). Crop genetic engineering: an approach to improve fungal resistance in plant system. *Plant-microbe interactions in agro-ecological perspectives*.

[8] Gaur R. K., Prajapat R., Marwal A., Sahu A., Rathore M. S. (2011). First report of a begomovirus infecting Mimosa pudica in India. *Journal of Plant Pathology*.

[9] Singh R., Srivastava A. (2020). Prevention and control of viral diseases of crops. *Applied Plant Virology: Advances, Detection and antiviral strategies*.

[10] Singh R., Raj S. K., Prasad V. (2008). Molecular characterization of a strain of squash leaf curl China virus from North India. *Journal of Phytopathology*.

[11] Singh R., Raj S. K., Chandra G. (2001). Association of a monopartite begomovirus with yellow mosaic disease of pumpkin (*Cucurbita maxima*) in India. *Plant Disease*.

[12] Raj S. K., Snehi S. K., Khan M. S., Singh R., Khan A. A. (2008). Molecular evidence for association of Tomato leaf curl New Delhi virus with leaf curl disease of papaya (Carica papaya L.) in India. *Australasian Plant Disease Notes*.

[13] Klinkowski M. (1970). Catastrophic plant diseases. *Annual Review of Phytopathology*.

[14] Lana A. F., Adegbola M. O. K. (1977). Important virus diseases in West African crops. *Review of Plant pathology*.

[15] Bos L. (1982). Crop losses caused by viruses. *Crop Protection*.

[16] Barnett O. W., Diachun S., Edwardson S. R., Cristie R. G. (1986). Virus diseases of clovers: etiology and crop losses. *Viruses Infecting Forage Legumes II*.

[17] Martelli G. P. (1986). Virus and virus-like diseases of the grapevine in the Mediterranean area. *FAO plant protection bulletin*.

[18] Godo G., Fauquet C., Fargette D. (1988). General synthesis of national reports on the African cassava mosaic and its control. *La mosaique du Manioc et son Control*.

[19] Lovisolo O., Caciagli P. (1989). Tentativi di valutazione dei danni causati alle colture da malattie virali e similvirali. *Informatore Fitopatologico*.

[20] Agrios G. N., Mandahar C. L. (1990). Economic considerations. *Plant Viruses. Vol. II. Pathology*.

[21] Roistacher C. N., Moreno P. The worldwide threat from destructive isolates of citrus tristeza virus. A review.

[22] Thottappilly G. (1992). Plant virus diseases of importance to African agriculture∗. *Journal of Phytopathology*.

[23] Rush C. M., Heidel G. B. (1995). Fuvovirus diseases of sugar beets in the United States. *Plant Disease*.

[24] Almeida R. P. P. (2018). Emerging plant disease epidemics: biological research is key but not enough. *PLoS Biology*.

[25] Nicaise V. (2014). Crop immunity against viruses: outcomes and future challenges. *Frontiers in plant science*.

[26] Liu R., Lal R. (2015). Potentials of engineered nanoparticles as fertilizers for increasing agronomic productions. *Science of the total environment*.

[27] Prasad R., Kumar V., Prasad K. S. (2014). Nanotechnology in sustainable agriculture: present concerns and future aspects. *African Journal of Biotechnology*.

[28] Tarafder C., Daizy M., Alam M. M. (2020). Formulation of a hybrid nanofertilizer for slow and sustainable release of micronutrients. *ACS Omega*.

[29] Elizabeth A., Worrall A. H., Karishma T., Mody N. M., Pappu H. R. (2018). Nanotechnology for Plant Disease Management. *Agronomy*.

[30] Khan I., Saeed K., Khan I. (2019). Nanoparticles: properties, applications and toxicities. *Arabian Journal of Chemistry*.

[31] Srivastava A., Singh R. (2021). Nanoparticles for sustainable agriculture and their effect on plants. *Current Nanoscience*.

[32] Khan A. A., Naqvi Q. A., Khan M. S., Singh R., Raj S. K. (2005). First report of a begomovirus infecting calendula in India. *Plant pathology*.

[33] Zhuang J., Gentry R. W. (2011). Environmental application and risks of nanotechnology: a balanced view. *Biotechnology and nanotechnology risk assessment: minding and managing the potential threats around us*.

[34] Loureiro A., Azoia N. G., Gomes A. C., Cavaco-Paulo A. (2016). Albumin-based nanodevices as drug carriers. *Current Pharmaceutical Design*.

[35] Nikalje A. P. (2015). Nanotechnology and its applications in medicine. *Medicinal Chemistry*.

[36] Srivastava A., Chuhan S. P., Singh (2021). Effect of silver nanoparticles on the growth and development of *Indian brassica* and *Cicer arietinum*. *Research Journal of Biotechnology*.

[37] Ripp S., Henry T. B. (2011). *Biotechnology and Nanotechnology Risk Assessment: Minding and Managing the Potential Treats around US*.

[38] Golobič M., Jemec A., Drobne D., Romih T., Kasemets K., Kahru A. (2012). Upon exposure to Cu nanoparticles, accumulation of copper in the isopod Porcellio scaber is due to the dissolved Cu ions inside the digestive tract. *Environmental Science and Technology*.

[39] Avasare V., Zhang Z., Avasare D., Khan I., Qurashi A. (2015). Room-temperature synthesis of TiO_2_ nanospheres and their solar driven photoelectrochemical hydrogen production. *International Journal of Energy Research*.

[40] Ning F., Shao M., Xu S. (2016). TiO_2_/graphene/NiFe-layered double hydroxide nanorod array photoanodes for efficient photoelectrochemical water splitting. *Energy & Environmental Science*.

[41] Wang Y., Sun C., Xu C. (2016). Preliminary experiments on nano-silver against tobacco mosaic virus and its mechanism. *Tobacco Science and Technology*.

[42] Sinha K., Ghosh J., Sil P. C., Mihai A. (2017). New pesticides: a cutting-edge view of contributions from nanotechnology for the development of sustainable agricultural pest control. *New Pesticides and Soil Sensors*.

[43] Balaure P. C., Gudovan D., Gudovan I. (2017). Nanopesticides: a new paradigm in crop protection. *New pesticides and soil sensors*.

[44] Elmer W., White J. C. (2018). The future of nanotechnology in plant pathology. *Annual Review of Phytopathology*.

[45] Ahmadian K., Jalilian J., Pirzad A. (2021). Nano-fertilizers improved drought tolerance in wheat under deficit irrigation. *Agricultural Water Management*.

[46] Ni D., Bu W., Ehlerding E. B., Cai W., Shi J. (2017). Engineering of inorganic nanoparticles as magnetic resonance imaging contrast agents. *Chemical Society Reviews*.

[47] Iravani S. (2011). Green synthesis of metal nanoparticles using plants. *Green Chemistry*.

[48] Mittal A. K., Chisti Y., Banerjee U. C. (2013). Synthesis of metallic nanoparticles using plant extracts. *Biotechnology advances*.

[49] Alghuthaymi M. A., Almoammar H., Rai M., Said-Galiev E., Abd-Elsalam K. A. (2015). Myconanoparticles: synthesis and their role in phytopathogens management. *Biotechnology & Biotechnological Equipment*.

[50] Datnoff L. E., Elmer W. H., Huber D. M. (2007). *Mineral Nutrition and Plant Disease*.

[51] Sanchez-Dominguez M., Boutonnet M., Solans C. (2009). A novel approach to metal and metal oxide nanoparticle synthesis: the oil-in-water microemulsion reaction method. *Journal of nanoparticle research*.

[52] Rajput V., Minkina T., Behal A. (2018). Effects of zinc-oxide nanoparticles on soil, plants, animals and soil organisms: a review. *Environmental Nanotechnology, Monitoring & Management*.

[53] Richards R. M. (1981). Antimicrobial action of silver nitrate. *Microbios*.

[54] Elbeshehy E. K., Elazzazy A. M., Aggelis G. (2015). Silver nanoparticles synthesis mediated by new isolates of *Bacillu*s spp., nanoparticle characterization and their activity against bean yellow mosaic virus and human pathogens. *Frontiers in microbiology*.

[55] Cordero T., Mohamed M. A., López-Moya J. J., Daròs J. A. (2017). A recombinant potato virus Y infectious clone tagged with the rosea1 visual marker (pvy–ros1) facilitates the analysis of viral infectivity and allows the production of large amounts of Anthocyanins in plants. *Frontiers in microbiology*.

[56] Hao Y., Cao X., Ma C. (2017). Potential applications and antifungal activities of engineered nanomaterials against gray mold disease agent Botrytis cinerea on rose petals. *Frontiers in Plant Science*.

[57] Kreibig U., Vollmer M. (1995). Optical properties of metal *clusters*. *Springer Series in Material Science*.

[58] Mulvaney P. (1996). Surface plasmon spectroscopy of nanosized metal particles. *Langmuir*.

[59] Morones J. R., Elechiguerra J. L., Camacho A. (2005). The bactericidal effect of silver nanoparticles. *Nanotech*.

[60] Lara H. H., Ayala-Núñez N. V., Ixtepan Turrent L. C., Rodríguez Padilla C. (2010). Bactericidal effect of silver nanoparticles against multidrug–resistant bacteria. *World Journal of Microbiology and Biotechnology*.

[61] Mehrbod P., Motamed N., Tabatabaian M., Soleimani Estyar R., Amini E., Shahidi M. (2009). In vitro antiviral effect of “nanosilver” on influenza virus. *Daru*.

[62] Borkow G., Gabbay J. (2004). Putting copper into action: copper–impregnated products with potent biocidal activities. *FASEB Journal*.

[63] Borkow G., Gabbay J. (2009). Copper, an ancient remedy returning to fight microbial, fungal and viral infections. *ACS Chemical Biology*.

[64] Baker C., Pradhan A., Pakstis L., Pochan D. J., Shah S. I. (2005). Synthesis and antibacterial properties of silver nanoparticles. *Journal of nanoscience and nanotechnology*.

[65] Ruparelia J. P., Chatterjee A. K., Duttagupta S. P., Mukherji S. (2008). Strain specificity in antimicrobial activity of silver and copper nanoparticles. *Acta Biomaterialia*.

[66] Rai M., Yadav A., Gade A. (2009). Silver nanoparticles as a new generation of antimicrobials. *Biotechnology Advances*.

[67] El-Dougdoug N. K., Bondok A. M., El-Dougdoug K. A. (2018). Evaluation of silver nanoparticles as antiviral agent against ToMV and PVY in tomato plants. *Sciences*.

[68] Jain D. (2014). Green synthesis of silver nanoparticles and their application in plant virus inhibition. *Journal of mycology and plant pathology*.

[69] Shafie R. M., Salama A. M., Farroh K. Y. (2018). Silver nanoparticles activity against tomato spotted wilt virus. *Middle East Journal of Agriculture Research*.

[70] El-shazly M., Attia Y., Kabil F., Anis E., Hazman M. (2017). Inhibitory effects of salicylic acid and silver nanoparticles on potato virus Y-infected potato plants in Egypt. *Middle East Journal of Agriculture Research*.

[71] Cai L., Liu C., Fan G., Liu C., Sun X. (2019). Preventing viral disease by ZnONPs through directly deactivating TMV and activating plant immunity in Nicotiana benthamiana. *Environmental Science: Nano*.

[72] Kochkina Z., Pospeshny G., Chirkov S. (1994). Inhibition by chitosan of productive infection of T-series bacteriophages in the Escherichia coli culture. *Mikrobiologiia*.

[73] Chirkov S. (2002). The antiviral activity of chitosan (review). *Applied Biochemistry and Microbiology*.

[74] Kashyap P. L., Xiang X., Heiden P. (2015). Chitosan nanoparticle based delivery systems for sustainable agriculture. *International journal of biological macromolecules*.

[75] Malerba M., Cerana R. (2016). Chitosan effects on plant systems. *International journal of molecular sciences*.

[76] Adeel M., Farooq T., White J. C., Hao Y., He Z., Rui Y. (2021). Carbon-based nanomaterials suppress tobacco mosaic virus (TMV) infection and induce resistance in Nicotiana benthamiana. *Journal of Hazardous Materials*.

[77] Zein H. S., Miyatake K. (2009). Development of rapid, specific and sensitive detection of cucumber mosaic virus. *African Journal of Biotechnology*.

[78] Sun W., Jiao K., Zhang S., Zhang C., Zhang Z. (2001). Electrochemical detection for horseradish peroxidase-based enzyme immunoassay using p-aminophenol as substrate and 12 its application in detection of plant virus. *Analytica Chimica Acta*.

[79] Zein H. S., da Silva J. A. T., Miyatake K. (2009). Antigenic properties of the coat of *Cucumber mosaic virus* using monoclonal antibodies. *Journal of virological methods*.

[80] Raj S. K., Singh R., Pandey S. K., Singh B. P. (2003). Association of geminivirus with a leaf curl disease of Sunn hemp (*Crotalaria juncea*) in India. *European journal of plant pathology*.

[81] Chartuprayoon N., Rheem Y., Ng J. C. K., Nam J., Chen W., Myung N. V. (2013). Polypyrrole nanoribbon based chemiresistive immunosensors for viral plant pathogen detection. *Analytical Methods*.

[82] Boonham N., Glover R., Tomlinson J., Mumford R., Collinge D. B. (2008). Exploiting generic platform technologies for the detection and identification of plant pathogens. *Sustainable Disease Management in a European Context*.

[83] Yao K. S., Li S. J., Tzeng K. C. (2009). Fluorescence silica nanoprobe as a biomarker for rapid detection of plant pathogens. *Advanced Materials Research*.

[84] Li X. M., Xu G., Liu Y., He T. (2011). Magnetic Fe3O4 nanoparticles: synthesis and application in water treatment. *Nanoscience & Nanotechnology-Asia*.

[85] Tartaj P., del Puerto M. M., Veintemillas-Verdaguer S., Gonzalez-Carreno T., Serna C. J. (2003). The preparation of magnetic nanoparticles for applications in biomedicine. *Journal of physics D: Applied physics*.

[86] Ahmadov S., Ramazanov M. A., Sienkiewicz A., Forro L. (2014). Uptake and intracellular trafficking of super paramagnetic iron oxide nanoparticles (SPIONs) in plants. *Digest Journal of Nanomaterials and Biostructures*.

[87] Cash K. J., Plaxco K. W. (2021). Signal transduction with a swing. *Nature Chemistry*.

[88] Perdikaris A., Vassilakos N., Yiakoumettis I., Kektsidou O., Kintzios S. (2011). Development of a portable, high throughput biosensor system for rapid plant virus detection. *Journal of virological methods*.

[89] James C. (2013). Polypyrrole nanoribbon based chemiresistive immunosensors for viral plant pathogen detection. *Analytical Methods*.

[90] Lin H. Y., Huang C. H., Lu S. H., Kuo I. T., Chau L. K. (2014). Direct detection of orchid viruses using nanorod-based fiber optic particle plasmon resonance immunosensor. *Biosensors and Bioelectronics*.

[91] Jarocka U., Wąsowicz M., Radecka H., Malinowski T., Michalczuk L., Radecki J. (2011). Impedimetric immunosensor for detection of *plum pox virus* in plant extracts. *Electroanalysis*.

[92] Jarocka U., Radecka H., Malinowski T., Michalczuk L., Radecki J. (2013). Detection of Prunus necrotic ringspot virus in plant extracts with impedimetric immunosensor based on glassy carbon electrode. *Electroanalysis*.

[93] Huang X., Xu J., Ji H.-F., Li G., Chen H. (2014). Quartz crystal microbalance based biosensor for rapid and sensitive detection of maize chlorotic mottle virus. *Analytical Methods*.

[94] Tsuda S., Mitsuro K.-I., Hanada K., Kouda Y., Hikata M., Tomaru K. (1992). Novel detection and identification technique for plant viruses: rapid immunofilter paper assay (RIPA). *Plant Disease*.

[95] Salomone A., Mongelli M., Roggero P., Boscia D. (2004). Reliability of detection of citrus tristeza virus by an immunochromatographic lateral flow assay in comparison with ELISA. *Journal of Plant Pathology*.

[96] Danks C., Barker I. (2000). On-site detection of plant pathogens using lateral-flow devices∗. *EPPO Bulletin*.

[97] Drygin Y. F., Blintsov A. N., Grigorenko V. G. (2012). Highly sensitive field test lateral flow immunodiagnostics of PVX infection. *Applied microbiology and biotechnology*.

[98] Kim J. H., Kim S. K., Wang K. C., Cho B. K., Tominaga T. (2010). Ischemia/angiogenesis-related molecules and cells. *Moyamoya disease update*.

[99] Mushaben E. M., Brandt E. B., Hershey G. K. K., Le Cras T. D. (2013). Differential effects of rapamycin and dexamethasone in mouse models of established allergic asthma. *PLoS One*.

[100] Charlermroj R., Himananto O., Seepiban C. (2013). Multiplex detection of plant pathogens using a microsphere immunoassay technology. *PLoS One*.

[101] Boltovets P. M., Boyko V. R., Kostikov I. Y., Dyachenko N. S., Snopok B. A., Shirshov Y. M. (2002). Simple method for plant virus detection: effect of antibody immobilization technique. *Journal of Virological Methods*.

[102] Torrance L., Ziegler A., Pittman H., Paterson M., Toth X., Eggleston I. (2006). Oriented immobilisation of engineered single-chain antibodies to develop biosensors for virus detection. *Journal of virological methods*.

[103] Skottrup P., Hearty S., Frøkiær H. (2007). Detection of fungal spores using a generic surface plasmon resonance immunoassay. *Biosensors and Bioelectronics*.

[104] Skottrup P., Nicolaisen M., Justesen A. F. (2007). Rapid determination of Phytophthora infestans sporangia using a surface plasmon resonance immunosensor. *Journal of microbiological methods*.

[105] Frasco M. F., Chaniotakis N. (2009). Semiconductor quantum dots in chemical sensors and biosensors. *Sensor*.

[106] Algar W. R., Krull U. J. (2008). Quantum dots as donors in fluorescence resonance energy transfer for the bioanalysis of nucleic acids, proteins, and other biological molecules. *Analytical and Bioanalytical Chemistry*.

[107] Rad F., Mohsenifar A., Tabatabaei M. (2012). Detection of Candidatus Phytoplasma aurantifolia with a quantum dots fret-based biosensor. *Journal of Plant Pathology*.

[108] Safarpour H., Safarnejad M. R., Tabatabaei M. (2012). Development of a quantum dots FRET-based biosensor for efficient detection ofPolymyxa betae. *Canadian Journal of Plant Pathology*.

[109] du Jardin P. (2015). Plant biostimulants: definition, concept, main categories and regulation. *Scientia horticulturae*.

[110] Vazquez-Hernandez C., Feregrino-Perez A. A., Perez-Ramirez I. (2019). Controlled elicitation increases steviol glycosides (SGs) content and gene expression-associated to biosynthesis of SGs in *Stevia rebaudiana* B. cv. Morita II. *Industrial Crops and Products*.

[111] Rastogi A., Zivcak M., Sytar O. (2017). Impact of metal and metal oxide nanoparticles on plant: a critical review. *Frontiers in chemistry*.

[112] Cai L., Liu C., Fan G., Liu C., Sun X. (2019). Preventing viral disease by ZnONPs through directly deactivating TMV and activating plant immunity inNicotiana benthamiana. *Environmental Science: Nano*.

[113] Farooq T., Adeel M., He Z. (2021). Nanotechnology and plant viruses: an emerging disease management approach for resistant pathogens. *ACS Nano*.

[114] Derbalah A. S. H., Elsharkawy M. M. (2019). A new strategy to control cucumber mosaic virus using fabricated NiO-nanostructures. *Journal of Biotechnology*.

[115] Hao Y., Yuan W., Ma C. (2018). Engineered nanomaterials suppress turnip mosaic virus infection in tobacco (Nicotiana benthamiana). *Environmental Science: Nano*.

[116] Tan B. L., Norhaizan M. E., Liew W. P. P., Sulaiman R. H. (2018). Antioxidant and oxidative stress: a mutual interplay in age-related diseases. *Frontiers in pharmacology*.

[117] Hao Y., Fang P., Ma C. (2019). Engineered nanomaterials inhibit Podosphaera pannosa infection on rose leaves by regulating phytohormones. *Environmental research*.

[118] Soares C., Pereira R., Fidalgo F., Faisal M., Saquib Q., Alatar A., Al-Khedhairy A. (2018). Metal-based nanomaterials and oxidative stress in plants: current aspects and overview. *Phytotoxicity of Nanoparticles*.

[119] Elsharkawy M. M., Mousa K. M. (2015). Induction of systemic resistance againstPapaya ring spot virus(PRSV) and its vectorMyzus *persicaebyPenicillium* simplicissimumGP17-2 and silica (SiO2) nanopowder. *International journal of pest management*.

[120] Vinković T., Novák O., Strnad M. (2017). Cytokinin response in pepper plants (*Capsicum annuum* L.) exposed to silver nanoparticles. *Environmental research*.

[121] Rojas M. R., Hagen C., Lucas W. J., Gilbertson R. L. (2005). Exploiting chinks in the plant’s armor: evolution and emergence of geminiviruses. *Annual Review of Phytopathology*.

[122] García-Arenal F., Zerbini F. M. (2019). Life on the edge: geminiviruses at the interface between crops and wild plant hosts. *Annual Review of Virology*.

[123] Tiwari A. K., Snehi S. K., Singh R., Raj S. K., Rao G. P., Sharma P. K. (2011). Molecular identification and genetic diversity among six Begomovirus isolates affecting cultivation of cucurbitaceous crops in Uttar Pradesh, India. *Archives of Phytopathology and Plant Protection*.

[124] Briddon R. W., Markham P. G. (2000). Cotton leaf curl virus disease. *Virus Research*.

[125] Malathi V. G., Radhakrishnan G., Varma A., Loebenstein G., Thottappilly G. (2003). Cotton. *Virus and Virus-like Diseases of Major Crops in Developing Countries*.

[126] Moffat A. S. (1999). Geminiviruses emerge as serious crop threat. *Science*.

